# Forest Fragmentation as Cause of Bacterial Transmission among Nonhuman Primates, Humans, and Livestock, Uganda

**DOI:** 10.3201/eid1409.071196

**Published:** 2008-09

**Authors:** Tony L. Goldberg, Thomas R. Gillespie, Innocent B. Rwego, Elizabeth L. Estoff, Colin A. Chapman

**Affiliations:** University of Illinois, Urbana, Illinois, USA (T.L. Goldberg, T.R. Gillespie, E.L. Estoff); Makerere University, Kampala, Uganda (T.L. Goldberg, I.B. Rwego, C.A. Chapman); McGill University, Montreal, Quebec, Canada (C.A. Chapman); Wildlife Conservation Society, Bronx, New York, USA (C.A. Chapman); 1 Current affiliation: University of Wisconsin, Madison, Wisconsin, USA.

**Keywords:** Nonhuman primates, zoonoses, domestic animals, ecology, epidemiology, Escherichia coli, Uganda, Africa, research

## Abstract

We conducted a prospective study of bacterial transmission among humans, nonhuman primates (primates hereafter), and livestock in western Uganda. Humans living near forest fragments harbored *Escherichia coli* bacteria that were ≈75% more similar to bacteria from primates in those fragments than to bacteria from primates in nearby undisturbed forests. Genetic similarity between human/livestock and primate bacteria increased ≈3-fold as anthropogenic disturbance within forest fragments increased from moderate to high. Bacteria harbored by humans and livestock were approximately twice as similar to those of red-tailed guenons, which habitually enter human settlements to raid crops, than to bacteria of other primate species. Tending livestock, experiencing gastrointestinal symptoms, and residing near a disturbed forest fragment increased genetic similarity between a participant’s bacteria and those of nearby primates. Forest fragmentation, anthropogenic disturbance within fragments, primate ecology, and human behavior all influence bidirectional, interspecific bacterial transmission. Targeted interventions on any of these levels should reduce disease transmission and emergence.

## Abstract 2

Anthropogenic disturbance increases bacterial transmission.

Infectious diseases transmitted among wild nonhuman primates, humans, and domestic animals pose a serious threat to wildlife conservation, human health, and animal health (*1*,*2*). For example, outbreaks of Ebola hemorrhagic fever and anthrax have caused epidemic deaths in apes and local humans in West Africa (*3*,*4*), and human paramyxoviruses have caused repeated deaths in chimpanzees in Côte d’Ivoire (*5*). Emerging pathogens such as these are now regarded as important drivers of primate population declines (*1*,*6*).

Although people and domestic animals have shared habitats with nonhuman primates (primates hereafter) for centuries, the dynamics of these interactions have changed dramatically over the last several decades. The destruction of tropical forests worldwide has imperiled many primates (*7*). Today, most primates live in remnant forest fragments and isolated protected areas within habitat mosaics of farmland, pastures, and human settlements (*8*,*9*).

Several studies have demonstrated that fragmentation of tropical forests reduces primate biodiversity and alters primate demographics and behavior (*10*,*11*). Fragmentation also alters patterns of gastrointestinal helminthic and protozoan infection in certain species (*12*–*14*). Whether host susceptibility, transmission dynamics, or a combination of these factors drive such trends remains unclear (*15*). The effects of fragmentation on the dynamics of pathogen transmission between primates and other species, including humans, are largely unexplored.

The goal of this study was to assess the effects of forest fragmentation on rates and patterns of bacterial transmission among wild primates, humans, and livestock, and to examine how anthropogenic and behavioral factors affect these rates and patterns across a fragmented forest landscape. We targeted *Escherichia coli,* a common, genetically diverse gastrointestinal bacterium transmitted by a variety of modes, including directly and through the environment (*16*,*17*). Virulent forms of *E. coli* are of considerable concern as emerging zoonoses (*17*,*18*), and benign forms of the bacterium provide a useful system for understanding the transmission dynamics of a range of microbes with similar biologic and epidemiologic characteristics (*19*,*20*).

By examining genetic relationships among *E. coli* isolates from humans, livestock, and 3 species of primates, we inferred rates of bacterial transmission among populations of these species living in or near 3 fragments that differed in their degrees of anthropogenic disturbance. Combining bacterial genetic data with surveys of local residents allowed us to identify behavioral and demographic risk factors affecting bacterial transmission between humans and primates.

## Materials and Methods

### Study Site

**Figure 1:**
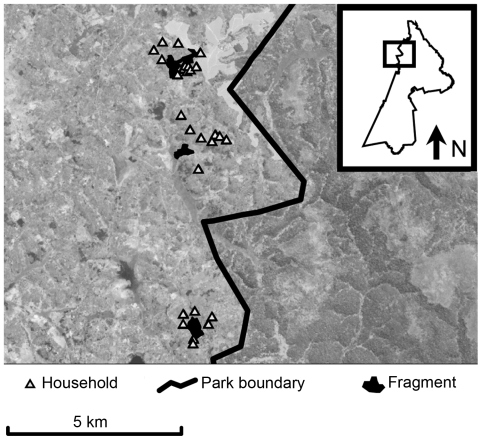
Map of study area within Kibale National Park, western Uganda (box) and forest fragments and households included in the study. Fragments are (from north to south) Kiko 1, Bugembe, Rurama (see Table 1 for details). Households, park boundary, and fragments are superimposed on a Landsat satellite image (30-m resolution).

The study took place in and near Kibale National Park, Uganda (Figure 1). Kibale is a 795-km^2^ park located in western Uganda near the foothills of the Rwenzori Mountains (0°13′–0°41′N, 30°19′–30°32′E), consisting primarily of moist semideciduous and evergreen forest, which is transitional between lowland rainforest and montane forest (elevation range ≈1,100–1,600 m) and interspersed with grassland, woodland, wetlands, and colonizing forest (*21*,*22*). Kibale is notable for its high species diversity and density of primates and is considered a premier primate research site in sub-Saharan Africa (*23*). Outside of the protected areas of Kibale exist a series of forest fragments that sustain small populations of primates (*11*). These fragments typically occupy nonarable wet lowlands. For this study, we focused on 3 fragments, Bugembe, Kiko 1, and Rurama, which have been studied intermittently since ≈1994 (Table 1).

**Table 1:** Characteristics of locations included in the study and sample sizes of households, persons, and *Escherichia coli* bacterial isolates*

Location	Characteristics						Sample sizes†					
	Area‡	Perimeter‡	Distance to park‡	Primate species present§	Disturbance¶		Household	Human	Livestock	BWC	RC	RT
Kibale National Park	792.73	197.20	0	BWC, RC, RT, + 9 others‡	Low		NA	NA	NA	10, 35	12, 46	7, 26
Bugembe fragment	0.66	1.48	1.69	BWC (11), RC (60), RT (10)	Medium		8	25, 52	27, 92	11, 42	9, 33	1, 4
Kiko 1 fragment	1.48	3.52	1.11	RC (4), RT (7)#	Very high		13	48, 139	16, 57	NA#	4, 13	4, 8
Rurama fragment	1.13	1.42	0.66	BWC (15), RC (15), RT (12)	High		7	26, 61	17, 53	13, 48	12, 46	10, 36

*BWC, black-and-white colobus (*Colobus guereza*); RC, red colobus (*Procolobus rufomitratus*); RT, red-tailed guenon (*Cercopithecus* *ascanius*); NA, not available. †Pairs of values indicate sample sizes of individuals and bacterial isolates, respectively. Household numbers indicate sample sizes of households enrolled in the study; approximately twice the indicated number of households are associated with each fragment. Although many households surround Kibale National Park, nonhuman primates were sampled only from core undisturbed forest sites where home ranges do not overlap with human settlements. Livestock included both cattle (*Bos indicus* and *B. taurus* x *B. indicus* crosses) and goats (*Caprus hircus*), which are combined here and in subsequent analyses because results were essentially identical when the species were analyzed separately. ‡Area (km^2^) and perimeter (km) were calculated by using the computer program ArcMap, version 9.1 (ESRI, Redlands, CA, USA), from point data gathered by walking the boundary of each fragment with a hand-held global positioning system unit. Distance to the park (km) was calculated as the shortest straight-line distance between the fragment and the park boundary. §Numbers in parentheses indicate population sizes of each species in each fragment in July 2005. See Struhsaker (*21*) for a description of the primate fauna and ecology of Kibale National Park. ¶Disturbance rankings are based on quantitative measures of encroachment from Onderdonk and Chapman (*11*) and from Gillespie and Chapman (*12*), as well as on qualitative assessments of forest clearing rates and intensities of human activity gathered from ground surveys in 2005–2006. #BWC had been extirpated from Kiko 1 fragment shortly before this study began, and RC were extirpated from the fragment shortly after sampling was completed, between January and July 2006. A combination of habitat destruction and hunting by domestic dogs led to the local extinctions of these 2 primate species.

### Study Species

We studied the 3 species of primates typically found in fragments near Kibale: red colobus (*Procolobus rufomitratus*), black-and-white colobus (*Colobus guereza*), and red-tailed guenons (*Cercopithecus ascanius*). The first 2 species are folivorous and can survive even on the depauperate forest vegetation of the fragments in which they reside (*11*). Red-tailed guenons are omnivorous primates that favor fruit and insects in undisturbed sections of Kibale (*24*) but survive in nearby fragments by habitually raiding crops from adjacent farmlands (*25*).

Small agricultural settlements surround each fragment (Table 1, Figure 1). Persons living in these settlements are primarily subsistence farmers. Their contact with primates occurs during excursions into fragments to extract forest resources (e.g., firewood, timber) or when primates leave fragments to raid crops. Primates must also cross pastures to move among disconnected habitats within fragments, thereby coming into close contact with livestock and their feces. Livestock included in this study were cattle (*Bos indicus* and *B. indicus* x *B. taurus* crosses) and goats (*Caprus hircus*), which are ubiquitous in the region. Humans and livestock in the region use common open water sources, such as open wells and streams, which tend to be located inside or near the edges of fragments, within primate home ranges.

### Sample Collection and Human Surveys

We collected fecal samples from primates (n = 93) during behavioral observations in June and July 2005 (dry season). We sampled all primate social groups from the 3 fragments as well as primates of the same species living in nearby undisturbed areas of Kibale National Park. Once a primate was observed to defecate, we recorded its species, age, sex, and individual identity (if known). We took care to sample only those portions of the fecal material that had not contacted the ground, to avoid environmental contamination. Environmental contamination from other sources (e.g., canopy vegetation) would have been unlikely, since we have been consistently unable to recover *E. coli* from such sources despite repeated attempts (T.L. Goldberg, unpub. data). Samples were placed in sterile tubes and transported within 6 hours to our field laboratory.

Maps and ground surveys were used to identify households within 0.5 km of each fragment; members of all of these households were invited to participate in the study in 2004. In June and July 2005, concurrent with primate sampling, members of each participating household (n = 99 persons) were given self-contained, sterile bacterial transport systems containing Cary-Blair agar (BD CultureSwab, Becton, Dickinson and Company, Franklin Lakes, NJ, USA) and were instructed in the proper method for self-administering a rectal swab. Inoculated swabs were collected and transported to our field laboratory within 24 hours of distribution. At the same time, fresh fecal samples from livestock (n = 60) owned by participating households were collected in sterile tubes and transported within 6 hours to our field laboratory.

At the time of human sample collection, a survey was administered to each participant. The survey focused on demographic information, personal health, patterns of forest use, and interactions with primates during the month before sample collection. The survey was administered in the local language by trained field assistants who were also members of the local communities; researchers were never present during survey administration to avoid response biases associated with the presence of foreigners. This study was reviewed and approved by the institutional review board and institutional animal care and use committee of the University of Illinois before data collection.

### Bacterial Isolation and Characterization

Swabs and fecal samples were streaked for isolation of *E. coli* onto individual MacConkey agar plates and incubated at 37°C for 24 hours in our field laboratory. Up to 6 putative *E. coli* colonies from each sample were transferred into tubes containing 0.1 mL tryptic soy agar and stored at room temperature for up to 4 weeks. Isolates were then shipped to the University of Illinois in the United States, re-isolated, subjected to standard biochemical tests for positive identification (*26*), and stored in 20% glycerol at –80°C for further analysis.

Confirmed *E. coli* isolates were genotyped by using Rep-PCR, which targets repetitive sequences dispersed throughout bacterial chromosomes (*27*). This method has high power for discriminating among *E. coli* isolates (*28*,*29*), and it can generate accurate phylogenetic information (*30*). DNA extraction, PCR, and electrophoresis protocols are described in detail elsewhere (*30*).

### Analyses

Rep-PCR genotypes were stored in the computer program BioNumerics, version 4.0 (Applied Maths, Austin, TX, USA). Relationships among isolates were inferred from Rep-PCR genotypes by using published methods that maximize the correspondence of such inferences to the standard of multilocus sequence typing (*30*). Population genetic analyses available in the computer program Arlequin, version 3.0 (*31*), were used to measure genetic differences among bacterial subpopulations. Specifically, analysis of molecular variance (AMOVA; *32*) was used to apportion genetic variation among different ecologically defined bacterial subpopulations, and the common genetic distance measure, *F_ST_* (*33*), was used to quantify short-term genetic distances among populations of bacteria from different host species and locations. Complementary phylogenetic analyses were conducted to examine relationships among individual bacterial isolates and to infer directional interspecific transmission; these were performed with BioNumerics and the computer programs PHYLIP, version 3.57c (*34*) and MacClade, version 4 (*35*), following a previously published analytical framework (*36*). Regression analyses were used to investigate the effects of putative demographic and behavioral risk factors on genetic similarity between bacteria from individual humans and bacteria from the primates inhabiting that person’s associated fragment (Appendix Table).

## Results

A total of 791 *E. coli* isolates from 252 individual persons, livestock, and primates were analyzed, representing (in the case of humans and livestock) 29 households (Table 1). Humans ranged in age from ≈2 months to 77 years and consisted of 48% male and 52% female participants. Sample sizes of primates were low in some locations, but this was inevitable, given small primate population sizes (Table 1). Human and livestock samples represented ≈50% of households surrounding each fragment.

**Figure 2:**
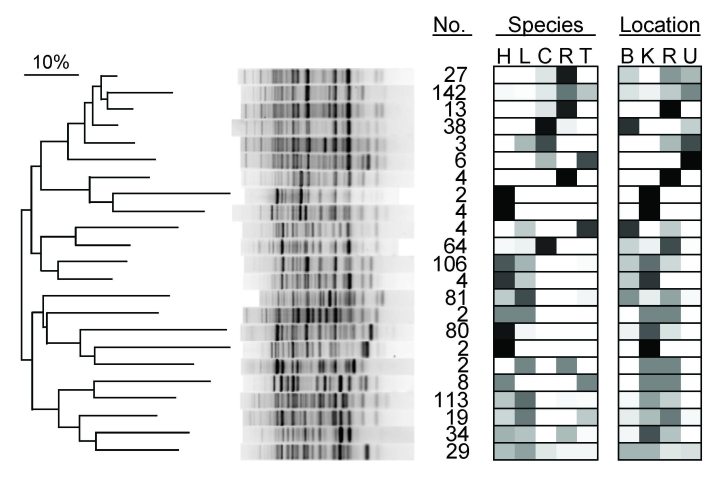
Dendrogram of genetic relatedness among 23 major clades of *Escherichia coli* from humans, domestic animals, and primates in 4 locations in and near Kibale National Park, western Uganda, derived from Rep-PCR genotypes. Major clades were identified from the full tree of 791 isolates by using the “cluster cutoff method” available in the computer program BioNumerics, version 4.0 (Applied Maths, Inc., Austin, TX, USA), which optimizes point-bisectional correlation across a range of cutoff similarity values to identify the most relevant clusters. A single representative bacterial genotype from each major clade is shown, and numbers of isolates falling within that clade are given (no.). Boxes indicate the host species and locations from which isolates in each clade were recovered and are shaded in proportion to the percentage of isolates in the clade from that species or location (0%, white; 100%, black). Species: H, human; L, livestock (cattle or goats); C, black-and-white colobus; R, red colobus; T, red-tailed guenon. Location: B, Bugembe fragment; K, Kiko 1 fragment; R, Rurama fragment; U, undisturbed locations within Kibale National Park. The dendrogram was drawn by using the neighbor-joining method (*37*) from a distance matrix generated from electrophoretic data that used optimized analytical parameters (*30*).

Phylogenetic analysis of bacterial genotypes identified 23 major clades (Figure 2), each containing between 2 and 142 unique genotypes. Some clades contained genotypes specific to particular species or locations; others contained genotypes from multiple species and multiple locations. Of the latter type, those containing isolates from both humans and primates tended to be phylogenetically clustered (Figure 2). Phylogenetic analyses of directional interspecific transmission (*36*) indicated no biases in transmission for different classes of directional transmission events (e.g., human to primate, primate to human). Analyses of molecular variance (Table 2) indicated that differences among species and locations accounted for only a small proportion of total bacterial genetic diversity (7.8% and 6.8%, respectively), and that individual fragments contained most bacterial genetic diversity (85.4%).

**Table 2:** Hierarchical analysis of molecular variance for *Escherichia coli* isolates collected from humans, nonhuman primates, and livestock associated with 3 forest fragments near Kibale National Park, western Uganda*

Variance component	Observed partition		φ statistic	p value†
	Variance	% Total		
Among species	0.672	7.76	φ*_CT_* = 0.078	<0.001
Among locations within species	0.592	6.84	φ*_SC_* = 0.074	<0.001
Within locations	7.395	85.41	φ*_ST_* = 0.146	<0.001

*Bacterial isolates were collected from 5 species in 3 locations. Locations were defined as Bugembe, Kiko 1, and Rurama fragments, and species were defined as humans, livestock (cattle and goats), black-and-white colobus, red colobus, and red-tailed guenons. Data consisted of bacterial genotypes represented as series of binary loci scored for the presence/absence of bands at each of 97 electrophoretic positions, by using the “bandmatch” procedure of the computer program BioNumerics, version 4.0 (Applied Maths, Inc., Austin, TX, USA) and optimized analytical parameters (*30*). Analysis of molecular variance was performed with the computer program Arlequin, version 3.0 (*31*). †Probability of having a more extreme variance component and φ statistic than the observed value by chance alone; probabilities were calculated from 16,000 random permutations of the data by using Arlequin, version 3.0 (*31*).

Pairwise bacterial genetic distances between metapopulations of primates, persons, and livestock are shown in Table 3. Both humans and livestock harbored bacteria significantly more similar genetically to those of primates in fragments than to those of primates in undisturbed locations within the national park. Humans and their livestock shared very similar bacteria, as indicated by an *F_ST_* of only 0.03; this genetic distance was smaller even than that between bacteria from primates in fragments and bacteria from primates in undisturbed forest (*F_S_* = 0.046), although this difference was not statistically significant.

**Table 3:** Matrix of pairwise interpopulation *F_ST_* values for *Escherichia coli* from humans, livestock, and nonhuman primates in Kibale National Park, western Uganda, and 3 nearby forest fragments

Bacterial population	*F_ST_* (SEM)*		
	Livestock	Nonhuman primates in forest fragments	Nonhuman primates in undisturbed forest
Humans			
Livestock	0.030 (0.007)^1^		
Nonhuman primates in forest fragments	0.102 (0.024)^2^	0.090 (0.021)^2^	
Nonhuman primates in undisturbed forest	0.180 (0.052)^3^	0.151 (0.051)^3^	0.046 (0.013)^1^

*F_ST_ values (which can vary between 0 and 1) represent short-term genetic distances between bacterial populations and were calculated from Rep-PCR data by using optimal analytical parameters (*30*). Standard errors were estimated from bootstrap analyses with 1,000 replicates. Each of the 6 *F_ST_* values shown is statistically significantly different from the null expectation of no genetic difference between populations, based on the bootstrap analysis (all p<0.01). Different superscript numbers indicate significantly different *F_ST_* values (exact probabilities <0.05).

**Figure 3:**
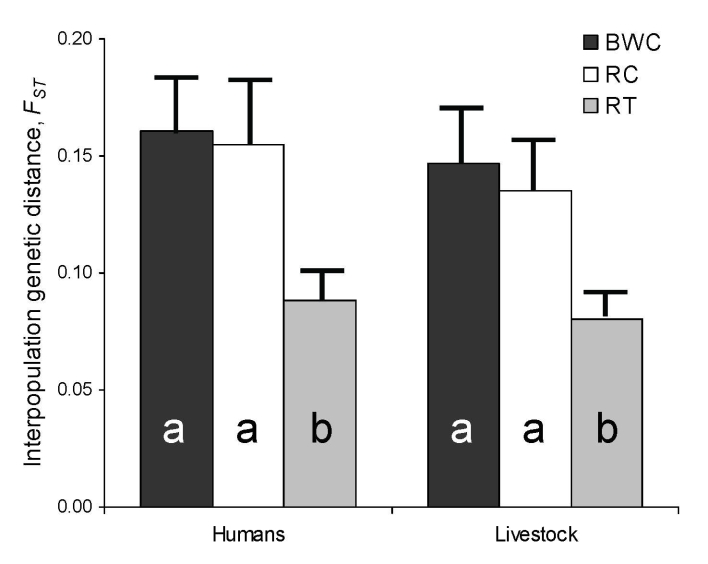
Interpopulation *F_ST_* values between *Escherica coli* from humans in villages associated with 3 forest fragments near Kibale National Park, Uganda, and *E. coli* from livestock and primates in the same village or fragment, respectively. *F_ST_* values between humans in each village and primates in undisturbed locations within Kibale National Park are shown for comparison. Error bars represent standard errors of the mean, estimated from bootstrap analyses with 1,000 replicates. Different letters within bars indicate statistically significantly different *F_ST_* values (exact probabilities <0.05).

**Figure 4:**
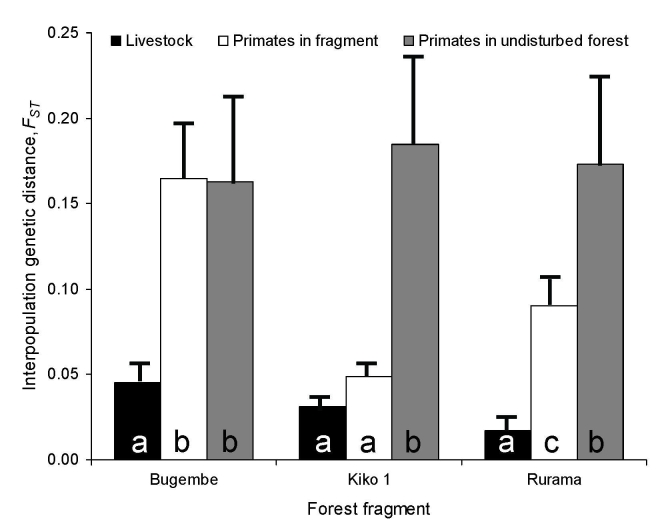
Interpopulation *F_ST_* values between *Escherichia coli* from 3 species of primates in 3 forest fragments near Kibale National Park, Uganda, and *E. coli* from both humans and livestock living in villages associated with the fragments. BWC, black-and-white colobus; RC, red colobus; RT, red-tailed guenon. Error bars represent standard errors of the mean, estimated from bootstrap analyses with 1,000 replicates. Different letters within bars indicate statistically significant differences in *F_ST_* values (exact probabilities <0.05).

Figure 3 shows the results of interspecies bacterial genetic distance analyses conducted separately for each fragment. Across fragments, bacteria from humans were uniformly genetically similar to bacteria from their livestock. However, genetic similarity between human and primate bacteria varied among fragments. Human–primate bacterial genetic similarity was highest in the Kiko 1 fragment, followed by Rurama, and then by Bugembe. This pattern parallels the relative degrees of anthropogenic disturbance of the fragments themselves (Kiko 1 >Rurama >Bugembe; Table 1). Species-specific analyses (Figure 4) indicated that bacteria from both humans and livestock were more similar to bacteria from red-tailed guenons than to bacteria from black-and-white colobus or red colobus.

Four variables were retained in the final regression model that examined associations between human behavioral predictors and human–primate bacterial genetic similarity (Appendix Table). Residence near a more disturbed fragment was the variable most strongly associated with increased genetic similarity between human and primate bacteria. Tending livestock, experiencing gastrointestinal symptoms, and fetching water from an open water source within the month before sampling were also associated with increased human–primate bacterial genetic similarity.

## Discussion

This study provides evidence that forest fragmentation increases bacterial transmission between primates and humans and their livestock. Bacteria from humans and livestock near 3 fragments were more similar genetically to bacteria from primates in those fragments than to bacteria of primates from nearby undisturbed forest locations. Moreover, the degree of disturbance of the fragments themselves paralleled the degree of genetic similarity between human and primate bacteria. Phylogenetic analyses and analyses of molecular variance further indicated that bacterial gene flow among species was high and that no directional biases in bacterial transmission were evident, findings that indicate that transmission of *E. coli* from primates to humans and livestock was as likely as transmission in the other direction.

Chapman et al. (*15*) recently showed that red colobus in forest fragments near Kibale suffer increased gastrointestinal parasitism with helminths as a result of nutritional stress and that this effect has led to a decline in population. Gillespie and Chapman (*12*) documented that the degree of disturbance of a fragment (measured as the density of tree stumps) was an accurate predictor of prevalence of infection of red colobus with parasitic nematodes. However, neither of these studies sampled humans or domestic animals, and neither examined transmission explicitly. The results of our study suggest that fragmentation may exert a heretofore-undocumented negative influence on the risk of primate infection by increasing pathogen transmission rates between primates and other species. Our results also show that the degree of anthropogenic disturbance within a fragment affects the rate at which bacteria are transmitted among species. Fragmentation likely leads to elevated interspecific transmission rates by increasing ecologic overlap among species.

The especially close genetic relationship between bacteria from humans and bacteria from red-tailed guenons (paralleled in livestock) probably reflects the propensity of red-tailed guenons to enter human habitats to raid crops (*25*). Unlike colobines, which can subsist on leaves even in highly degraded fragments, red-tailed guenons, which eat a more varied diet consisting of a high proportion of fruits, are likely obligate crop raiders in fragments. The importance of this species as a crop raider is evident from the fact that persons in our study area engage in a variety of culturally unique practices specifically designed to deter crop raiding, especially of maize, by red-tailed guenons (*38*).

We initially suspected that diet and digestive physiology might influence the genetic similarity of *E. coli* among different host species (*39*), but our results do not support this hypothesis. Humans in our study, who as omnivores have single stomachs, harbored *E. coli* virtually indistinguishable genetically from the *E. coli* of their cattle and goats, which are herbivores with chambered stomachs. By extension, similarities in digestive physiology between humans and red-tailed guenons would not be sufficient to account for the close genetic relationship between *E. coli* from these species. We infer that spatial and ecologic overlap is the primary determinant of bacterial genetic similarity among populations of hosts in our system.

Persons who tended livestock and experienced gastrointestinal symptoms during the month before sampling harbored bacteria genetically similar to those of the primates in their associated fragment, whereas persons who did not engage in these activities or experience these symptoms harbored bacteria more distantly related to those of the same primates. Tending livestock, which are often grazed in or near fragments, may bring humans into close contact with primates. Fetching water from an open water source (p = 0.07 in our regression analysis; see Appendix Table) may expose humans to water contaminated with bacteria of primate origin. We note that these variables accounted together for only 28% of variation in human–primate bacterial genetic similarity, indicating that most variation in this parameter remains unexplained.

We emphasize that the results of our risk analysis represent statistical associations and that they do not indicate direction of causality. For example, persons who experience gastrointestinal symptoms such as diarrhea may shed bacteria at high rates and thus be at increased risk of transmitting bacteria to primates; alternatively, persons who ingest microbes from primates might tend to experience gastrointestinal symptoms as a result. We also caution that our results might differ for pathogens more virulent than *E. coli*. For example, gastrointestinal disease would increase shedding of pathogens into the environment and affect host behavior. Our results are best interpreted as reflecting background patterns of bacterial transmission in the absence of confounders such as high virulence. Finally, we caution that assumptions inherent in our statistical analyses could affect the strength of the trends we have documented. For example, our analyses of genetic correspondence assume that parameters derived from maximum likelihood estimation are globally optimal, and our analyses of interpopulation genetic distances assume neutral molecular evolution (*33*).

We suspect that the patterns of bacterial genetic similarity we have documented reflect indirect transmission of microbes through the physical environment, such as through contaminated soil or water, rather than transmission by direct contact. For example, primates in the forest fragments near Kibale must come to the ground to cross open fields (often pastures) between habitat patches or to raid crops; this would increase their probability of encountering bacteria of human or livestock origin. Similarly, the location of fragments in nonarable, wet lowlands creates ideal conditions for contamination of surface water with primate feces. Unfortunately, our varied attempts to recover *E. coli* from water, soil, and vegetation were largely unsuccessful, perhaps because of the heat and aridity of the western Ugandan dry season (*40*).

Zoonotic diseases with primate origins have had global effects on human health (*1*). In Uganda, a high prevalence of HIV renders a significant proportion of the population immunocompromised and thus susceptible to opportunistic infections. Countries like Uganda are also undergoing rapid demographic changes and correspondingly rapid changes in land use. Our finding that a land-use change (forest fragmentation) enhances bacterial transmission between primates and an immunocompromised human population raises concerns about the potential for epidemics of zoonotic disease to originate from disturbed ecosystems such as this. Forest fragmentation may, in other words, negatively affect human public health by increasing the risks for zoonotic disease transmission from animals in forest fragments.

Forests and the primates living in them are disappearing rapidly from this region of Africa, which unfortunately typifies locations throughout the Tropics. We have already documented the extinction of 2 primate species from 1 fragment, and we predict that, without intervention, all unprotected fragments and their primates will disappear from our study area within the next 2 decades. Our results indicate that extinction of local primates may be accompanied by “spikes” in anthroponotic and zoonotic disease transmission risk, which could threaten not only the health of other primates and conservation but also human health. Mitigating these risks could entail such interventions as building closed wells, managing the grazing patterns of livestock, and encouraging the persistence of primate food trees within fragments. Understanding in greater detail how forest fragmentation and associated land-use changes affect pathogen transmission among primates, humans, and domestic animals would be critical for designing rational intervention strategies to conserve wild primates, as well as to safeguard human and animal health.

## Supplementary Material

Appendix Table Results of multivariate regression analysis of putative behavioral and demographic risk factors as predictors of genetic distance (FST) between bacteria from humans living in association with 3 forest fragments near Kibale National Park, western Uganda, and bacteria from primates living in the same forest fragment*
