# Erratum—Vol. 14, No. 9

**DOI:** 10.3201/eid1410.071196

**Published:** 2008-11

In Forest Fragmentation as Cause of Bacterial Transmission among Primates, Humans, and Livestock, Uganda (T.L. Goldberg et al.), 2 errors occurred. In Table 3, the numerical values are not in the right positions. The corrected table is available from www.cdc.gov/EID/content/14/9/1375-T3.htm. In the same article, Figures 3 and 4 were inadvertently reversed. This has also been corrected in the online version of the article (available from www.cdc.gov/EID/content/14/9/1375.htm).

