# Supplementary material for: Forest Fragmentation as Cause of Bacterial Transmission among Nonhuman Primates, Humans, and Livestock, Uganda
Source: Emerg Infect Dis. 2008 Sep;14(9):1375–82. doi: 10.3201/eid1409.071196 (PMC2603117; doi:10.3201/eid1409.071196)
Supplement: Appendix Table — Results of multivariate regression analysis of putative behavioral and demographic risk factors as predictors of genetic distance (FST) between bacteria from humans living in association with 3 forest fragments near Kibale National Park, western Uganda, and bacteria from primates living in the same forest fragment* [file 07-1196_appT-s1.pdf]

Appendix Table. Results of multivariate regression analysis of putative behavioral and demographic risk factors as predictors of genetic distance (F_{ST}) between bacteria from humans living in association with 3 forest fragments near Kibale National Park, western Uganda, and bacteria from primates living in the same forest fragment*

Variable†	β (SE)‡	sr^2-II§	*t* value	p value¶
Location	–0.053 (0.013)	12.48	–3.96	<0.001
Experienced gastrointestinal symptoms	–0.045 (0.019)	4.69	–2.42	0.009
Tended livestock	–0.044 (0.018)	4.62	–2.41	0.009
Fetched water from an open water source	–0.027 (0.018)	1.74	–1.48	0.071

*Variables were initially entered into a global multiple regression analysis and were removed individually to assess each variable's contribution to goodness-of-fit. The analysis was then repeated by using stepwise addition. Results were the same in both cases: variables not retained in the final model (see below) were clearly nonsignificant, as evidenced by p values all >0.49. Regression models were run, and standard regression model assumptions were tested by using the computer program SAS, version 9 (SAS Institute, Cary, NC, USA).

†Variables included in the table are those retained in the final regression model:

F_{ST} = 0.2 + (Location * –0.053) + (GI symptoms * –0.045) + (Tended livestock * –0.044) + (Fetched water * –0.027)

These variables together explained 27.5% of total variation in F_{ST} (R^2 = 0.275; F = 6.89; p<0.0001). Variables initially examined included the following (type of variable in parentheses), all of which pertained to the status of the participant during the 1-month period before sample collection, derived from interview data:

1. Age (interval): Age (y) of participant. Categorical variables classifying participants into age categories relevant to the sociodemographics of the region (0–1, 2–6, 7–16, 17–-25, >25) were also examined but were not significant in subsequent analyses and are therefore not included.
2. Collecting forest products (categorical): Whether or not a participant reported collecting any forest product (e.g., firewood, medicinal plants) (yes or no).
3. Experienced gastrointestinal symptoms (categorical): Whether or not a participant reported gastrointestinal symptoms (vomiting, diarrhea, cramps, or other indices of gastrointestinal upset) (yes or no).
4. Fetched water from an open water source (categorical): Whether or not a participant reported collecting water from an unprotected water source such as a stream or open well, as opposed to a closed pump (yes or no).
5. Guarding crops against crop raiding (categorical): Whether or not a participant reported guarding crops against raiding by wildlife, which encompasses a variety of activities that potentially increase direct contact with primates (yes or no).
6. Location (orthogonal contrast): Contrasts residence near a highly disturbed fragment (Kiko 1 or Rurama) with residence near a moderately disturbed fragment (Bugembe). A similar variable contrasting Kiko 1 with Rurama was not significant in subsequent analyses and is therefore not included.
7. Sex (categorical): Sex of participant (male or female).
8. Tended livestock (categorical): Whether or not a participant reported tending cattle or goats (yes or no).
9. Washing hands prior to eating (categorical): Whether or not a participant reported washing hands regularly before eating (yes or no).
10. Working in fields (categorical): Whether or not a participant reported engaging in agricultural fieldwork (yes or no).

‡β values (slopes, ± standard errors [SE]) indicate the amount of change in genetic distance (F_{ST}) between human and primate bacteria associated with a unit change in the independent variable. For example, having tended livestock within the month before sampling was, on average, associated with a reduction in human–primate genetic distance of 4.4 ± 1.8%.

§Squared semipartial correlation coefficient type II (sr^2-II) indicates the proportion of variance (%) in the dependent variable (genetic distance between bacteria, measured as F_{ST}) uniquely accounted for by each independent variable.

¶p values are 1-tailed; each of the factors retained in the final model was associated with the dependent variable in the predicted negative direction.
